# Application of Genetic Algorithm to Predict Optimal Sowing Region and Timing for Kentucky Bluegrass in China

**DOI:** 10.1371/journal.pone.0131489

**Published:** 2015-07-08

**Authors:** Erxu Pi, Liqun Qu, Xi Tang, Tingting Peng, Bo Jiang, Jiangfeng Guo, Hongfei Lu, Liqun Du

**Affiliations:** 1 College of Life and Environmental Science, Hangzhou Normal University, Hangzhou, Zhejiang Province, PR China; 2 College of Life Science, Zhejiang Sci-Tech University, Hangzhou, PR China; 3 College of Biology and Food Engineering, Changshu Institute of Technology, Changshu, PR China; 4 Meteorological Bureau of Shaanxi Province, Xi’an, Shaanxi Province, PR China; Instituto de Agricultura Sostenible (CSIC), SPAIN

## Abstract

Temperature is a predominant environmental factor affecting grass germination and distribution. Various thermal-germination models for prediction of grass seed germination have been reported, in which the relationship between temperature and germination were defined with kernel functions, such as quadratic or quintic function. However, their prediction accuracies warrant further improvements. The purpose of this study is to evaluate the relative prediction accuracies of genetic algorithm (GA) models, which are automatically parameterized with observed germination data. The seeds of five *P*. *pratensis* (Kentucky bluegrass, KB) cultivars were germinated under 36 day/night temperature regimes ranging from 5/5 to 40/40°C with 5°C increments. Results showed that optimal germination percentages of all five tested KB cultivars were observed under a fluctuating temperature regime of 20/25°C. Meanwhile, the constant temperature regimes (e.g., 5/5, 10/10, 15/15°C, etc.) suppressed the germination of all five cultivars. Furthermore, the back propagation artificial neural network (BP-ANN) algorithm was integrated to optimize temperature-germination response models from these observed germination data. It was found that integrations of GA-BP-ANN (back propagation aided genetic algorithm artificial neural network) significantly reduced the Root Mean Square Error (RMSE) values from 0.21~0.23 to 0.02~0.09. In an effort to provide a more reliable prediction of optimum sowing time for the tested KB cultivars in various regions in the country, the optimized GA-BP-ANN models were applied to map spatial and temporal germination percentages of blue grass cultivars in China. Our results demonstrate that the GA-BP-ANN model is a convenient and reliable option for constructing thermal-germination response models since it automates model parameterization and has excellent prediction accuracy.

## Introduction

Seed germination rate is often used to evaluate the suitability of an environment for the cultivation of different plant species [1–3]. Constructing a mathematical model that accurately predicts the effect of temperature on germination percentage helps to reduce failure of grass establishment caused by inappropriate sowing dates or mismatching of grass species with climate zones; therefore it is particularly useful in selecting appropriate grass species and sowing times. Several mathematic models have been developed to simulate the germination response to temperature based on the experimental data [4–9]. These previous models were mainly used to predict: I. the time, under a constant temperature condition (cumulative temperature), required for the expected germination of a specific variety [4–6], and II. the germination percentage under a temperature fluctuation regime [10–12]. It is well-documented that the natural fluctuations in temperature between day and night could be required for initiating and/or facilitating seed germination [8], these diurnal fluctuations of temperature are frequently adopted to generate data for building prediction models [13]. To date, the core functions of the published temperature-germination response models [11,12] consist on the estimation of populations’ thermal response parameters [14–18] or on optimization of polynomial equations using iterative curve fitting [6,10]. These functions constructed by various scientists are usually different from each other because their parameters are selected for fitting the germination data of a particular batch of seeds. In addition, the different researchers’ preferences on selections of core functions and parameters for fitting the germination models might generate different outputs. Since the seed germination is influenced by various factors, scientists should develop multi-objective evolutionary algorithms for the germination response model. Recent years, the genetic algorithm (GA) is widely used as a non-dominated sorting based multi-objective evolutionary approach that doesn’t need specifying a set of sharing parameters [19–21]. In an effort to simplify and standardize the selection of core function for a temperature-germination model, we propose a GA-based data mining approach that automatically generates a core function of seed germination and temperature correlation. Meanwhile, the back propagation (BP) algorithm is also recruited to optimize the GA based temperature-germination model [10].

*Poa pratensis* L. (Kentucky bluegrass, KB) has long been used in lawns because of its excellent agronomic characteristics [22–25]. Compared to other winter-season turfgrass species, this species has fairly low irrigation requirements due to its good tolerance to drought stress [26–28]. It is also adapted to a moderate range of salinity and alkali stresses [29–32]. Though the KB could survive a wide range of temperature conditions, its germination is very sensitive to extreme temperatures [33–36]. Hence, the KB would be an ideal species for optimizing temperature-germination response models. In addition, the optimized models would directly help decision-maker in selecting optimal sowing regions and times for this broadly cultivated grass. To further facilitate KB cultivation in China, the GIS temperature data covering the whole nation were used to generate accurate and quantitative suitability maps for cultivation of KB cultivars, which have already been imported and are quickly gaining acceptance to many areas in the country [37,38]. Briefly, the means of minimum and maximum temperature data on the national geo-grid of China for a 25-years period, obtained from NASA, were used for calculating suitability values of the tested cultivar in the optimized GA-BP-ANN temperature-germination prediction functions.

The objectives of this paper are to: (i) provide an automatic approach (GA-BP-ANN) on revealing the seed temperature-germination relationships, (ii) use the GA-BP-ANN based temperature-germination models to predict the suitability of five KB cultivars (‘Midnight II’, ‘Diva’, ‘Rugby II’, ‘Leopard’ and ‘Sapphire’) throughout the national temperature grids in China. In short, it is tried to construct a new approach for grass suitability evaluation, and provide decision-maker with some useful information for selecting reasonable sowing regions and times for KB.

## Results

### Germination response to diurnal fluctuations of temperature

The germination responses of the five tested KB cultivars were similar to each other (Tables 1–5), and the mean germination percentages under all the tested temperature regimes showed no significant difference for all the five tested cultivars (P > 0.05). They could all germinate at cool-period temperatures ranging from 10 to 30^°C^ combined with warm-period temperatures ranging from 15 to 35^°C^. Considering optimum germination is usually defined as a germination percentage of not lower than the maximum germination minus one-half of its confidence interval (P = 0.05), optimum germination was found to be reached when the tested seeds were grown under a temperature regime with a cool-temperature between 10~25°C combined with a warm-temperature between 25~35°C. The germination percentages of all five cultivars were lower than 50% under constant temperatures within the thermal range from 10 to 30°C, while no germination was registered at constant 35°C. The maximum germination was observed when the warm-temperature was 5~10°C above the cool-temperature of the fluctuating thermal regime (Tables 1–5). In other words, the fluctuating temperature regimes in the range from 15 to 35°C promote KB germination.

**Table 1:** Cumulative seed germination of ‘Midnight II’ at different days in 36 temperature regimes (50 seeds in total).

Cool period temperature (°C) 16h	Warm period temperature (°C) 8h							
	5	10	15	20	25	30	35	40
	% Germination after 15–20 days							
5	0.0±0.0	0.0±0.0	0.0±0.0	6.0±4.0	50.7±5.0	43.3±9.0	0.0±0.0	0.7±1.2
10		6.0±0.0	16.7±2.3	28.0±2.0	66.7±4.3	74.0±3.5	0.0±0.0	0.0±0.0
15			25.3±7.0	46.0±6.9	78.0±6.0	72.0±7.2	34.0±6.0	0.0±0.0
20				23.3±9.5	80.7±1.2	81.3±8.1	64.0±2.0	0.0±0.0
25					18.7±1.3	78.7±10.3	68.7±5.8	0.0±0.0
30						38.7±9.0	24.7±4.6	0.0±0.0
35							0.0±0.0	0.0±0.0
40								0.0±0.0

**Table 2:** Cumulative seed germination of ‘Diva’ at different days in 36 temperature regimes (50 seeds in total).

Cool period temperature (°C) 16h	Warm period temperature (°C) 8h							
	5	10	15	20	25	30	35	40
	% Germination after 15–20 days							
5	0.0±0.0	0.0±0.0	0.0±0.0	6.7±1.2	44.7±2.3	44.0±8.7	0.0±0.0	0.0±0.0
10		6.0±0.0	13.3±4.2	35.3±3.1	77.3±3.1	73.3±17.0	0.0±0.0	0.0±0.0
15			14.0±2.0	52.7±4.2	79.3±6.4	82.0±5.3	40.0±3.5	0.0±0.0
20				20.0±2.0	82.0±5.3	81.3±8.1	69.3±8.1	0.0±0.0
25					26.0±4.0	78.0±8.0	53.3±4.2	0.0±0.0
30						35.3±7.0	38.7±3.1	0.0±0.0
35							0.0±0.0	0.0±0.0
40								0.0±0.0

**Table 3:** Cumulative seed germination of ‘Rugby II’ at different days in 36 temperature regimes (50 seeds in total).

Cool period temperature (°C) 16h	Warm period temperature (°C) 8h							
	5	10	15	20	25	30	35	40
	% Germination after 15–20 days							
5	0.0±0.0	0.0±0.0	0.0±0.0	10.7±3.1	44.0±7.2	52.7±8.1	0.0±0.0	0.0±0.0
10		6.0±2.0	8.7±3.1	38.0±8.0	63.3±3.1	69.3±2.3	0.0±0.0	0.0±0.0
15			19.3±3.1	58.7±2.3	70.7±5.0	80.7±9.2	44.0±2.0	0.0±0.0
20				27.3±1.2	64.7±25.0	87.3±3.1	76.0±8.0	0.0±0.0
25					22.0±2.0	79.3±5.0	46.0±2.0	0.0±0.0
30						33.3±4.2	52.0±7.2	0.0±0.0
35							0.0±0.0	0.0±0.0
40								0.0±0.0

**Table 4:** Cumulative seed germination of ‘Leopard’ at different days in 36 temperature regimes (50 seeds in total).

Cool period temperature (°C) 16h	Warm period temperature (°C) 8h							
	5	10	15	20	25	30	35	40
	% Germination after 15–20 days							
5	0.0±0.0	0.0±0.0	0.0±0.0	4.0±2.0	45.3±4.2	47.3±3.1	0.0±0.0	0.0±0.0
10		6.0±0.0	10.0±0.0	38.0±6.0	65.3±5.0	61.3±5.0	0.0±0.0	0.0±0.0
15			24.7±6.1	58.0±10.0	77.3±7.0	80.0±3.5	46.7±11.7	0.0±0.0
20				26.0±2.0	80.7±2.3	85.3±3.1	61.3±7.0	0.0±0.0
25					16.0±2.0	79.3±5.0	51.3±6.4	0.0±0.0
30						33.3±8.3	43.3±7.0	0.0±0.0
35							0.0±0.0	0.0±0.0
40								0.0±0.0

**Table 5:** Cumulative seed germination of ‘Sapphire’ at different days in 36 temperature regimes (50 seeds in total).

Cool period temperature (°C) 16h	Warm period temperature (°C) 8h							
	5	10	15	20	25	30	35	40
	% Germination after 15–20 days							
5	0.0±0.0	0.0±0.0	0.0±0.0	18.0±3.5	50.7±1.2	49.3±4.6	0.0±0.0	0.0±0.0
10		5.3±3.1	11.3±6.1	33.3±4.2	68.7±6.1	67.3±7.0	0.0±0.0	0.0±0.0
15			15.3±3.1	50.7±5.0	84.7±6.4	80.7±5.0	50.7±11.7	0.0±0.0
20				26.0±2.0	78.7±10.3	85.3±6.1	60.0±5.3	0.0±0.0
25					19.3±3.1	78.0±8.0	55.3±7.0	0.0±0.0
30						24.7±11.0	27.3±15.3	0.0±0.0
35							0.7±1.2	0.0±0.0
40								0.0±0.0

The means of the maximum and optima germination percentages of all the five KB cultivars are presented in Table 6. Maximum germination for ‘Leopard’ was the highest (87.3%), but not significantly different (P > 0.05) from that of the other four cultivars (81.3%, 82%, 85.3% and 85.3% for ‘Midnight II’, ‘Diva’, ‘Rugby II’ and ‘Sapphire’ respectively). Depending on grass cultivar, 11.1~22.2% (4~8 regimes) of the tested temperature regimes supported optimum germination. Only four tested regimes supported optimum germination for all the tested cultivars; they are 20/25, 15/30, 20/30, and 25/30°C (Tables 1–6).

**Table 6:** Comparison of the temperature–germination profiles for the five bluegrass cultivars.

Germination parameter	Sources				
	Midnight II	Diva	Rugby II	Leopard	Sapphire
	%				
Profile mean	28.5	29.2	28.9	29.3	28.6
Regimes with some germination	63.9	61.1	63.9	61.1	61.1
Maximum germination	81.3	82.0	85.3	87.3	85.3
Mean of some germination	44.6	47.8	45.3	47.9	46.8
Mean of optima	76.2	77.8	81.5	78.0	80.5

### Performance of different temperature-germination response models

The performances of GA-BP-ANN temperature-germination response models generated in this study were compared with the previously published regression approaches including general quadratic and BP-ANN based quintic equations [10, 12].

The RMSE values, which is proposed as statistical indicators for the evaluation and comparison of multi-dimensional models [39,40], also present similar performances among different KB cultivars (Table 7). For every KB cultivar, the RMSE values: GA-BP-ANN < BP-quintic < General quintic < BP-quadratic < General quadratic models. It suggested that GA-BP-ANN models present the best fitness for simulating the temperature-germination response of the tested five KB cultivars. In addition, the back propagation (BP) algorithm is an effective optimization tool for the tested non-linear regression models.

**Table 7:** The RMSE (Root Mean Square Error) values of different models for predicting temperature-germination response functions of the five *P*. *Pratenis* cultivars tested.

Cultivar	Model	RMSE
Midnight II	General quadratic	0.21
	BP-ANN quadratic	0.21
	Quintic	0.11
	BP-ANN quintic	0.08
	GA-BP-ANN	0.02
Diva	General quadratic	0.21
	BP-ANN quadratic	0.21
	Quintic	0.09
	BP-ANN quintic	0.07
	GA-BP-ANN	0.02
Rugby II	General quadratic	0.21
	BP-ANN quadratic	0.20
	Quintic	0.11
	BP-ANN quintic	0.10
	GA-BP-ANN	0.09
Leopard	General quadratic	0.23
	BP-ANN quadratic	0.21
	Quintic	0.08
	BP-ANN quintic	0.07
	GA-BP-ANN	0.06
Sapphire	General quadratic	0.21
	BP-ANN quadratic	0.20
	Quintic	0.07
	BP-ANN quintic	0.07
	GA-BP-ANN	0.02

ANN: Artificial Neural Network.

### Spatial mapping of optimal sowing times

**Fig 1:**
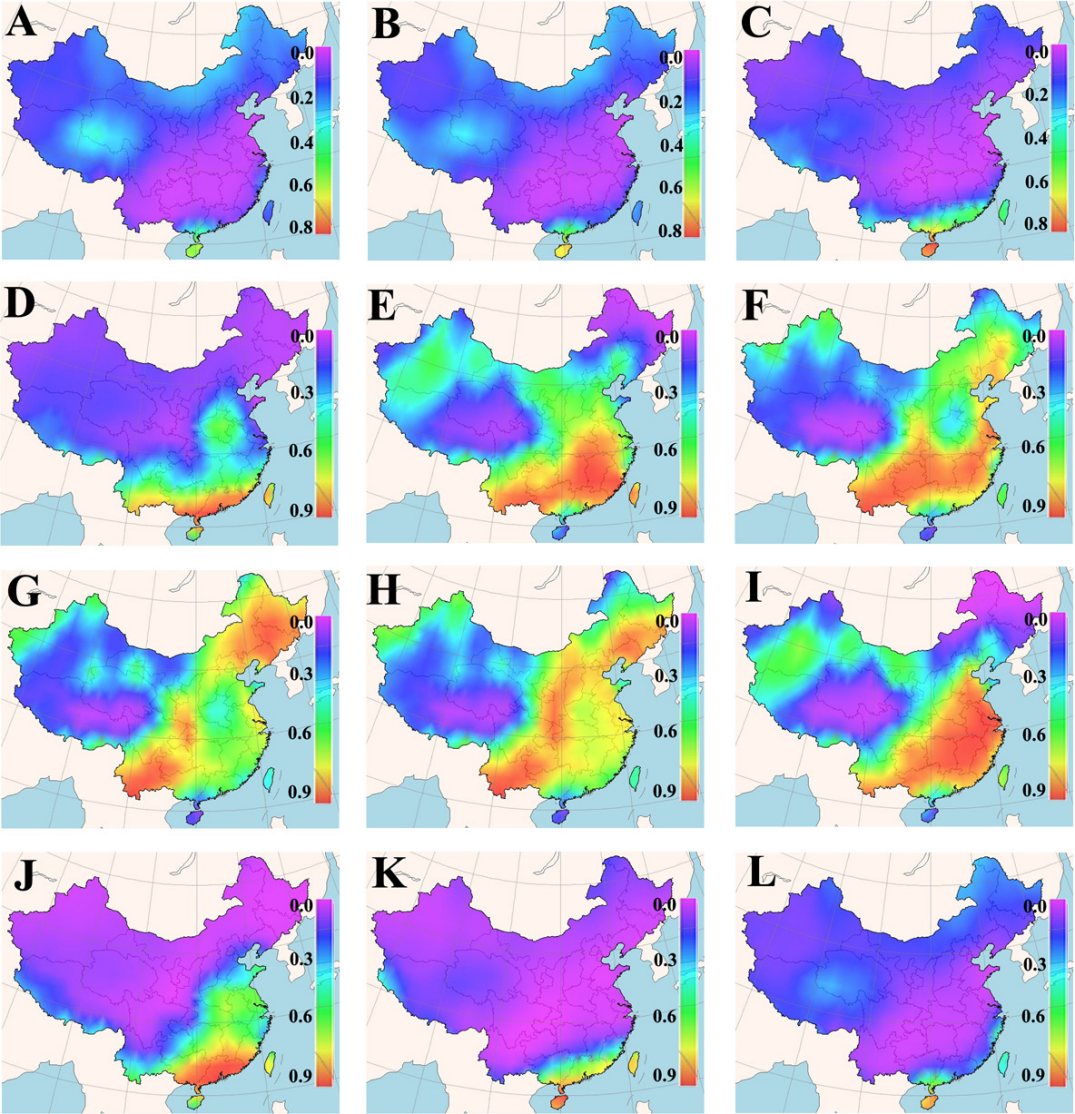
Maps of monthly germination suitability of ‘Midnight II’ in different regions of China; (A-L) January–December.

**Fig 2:**
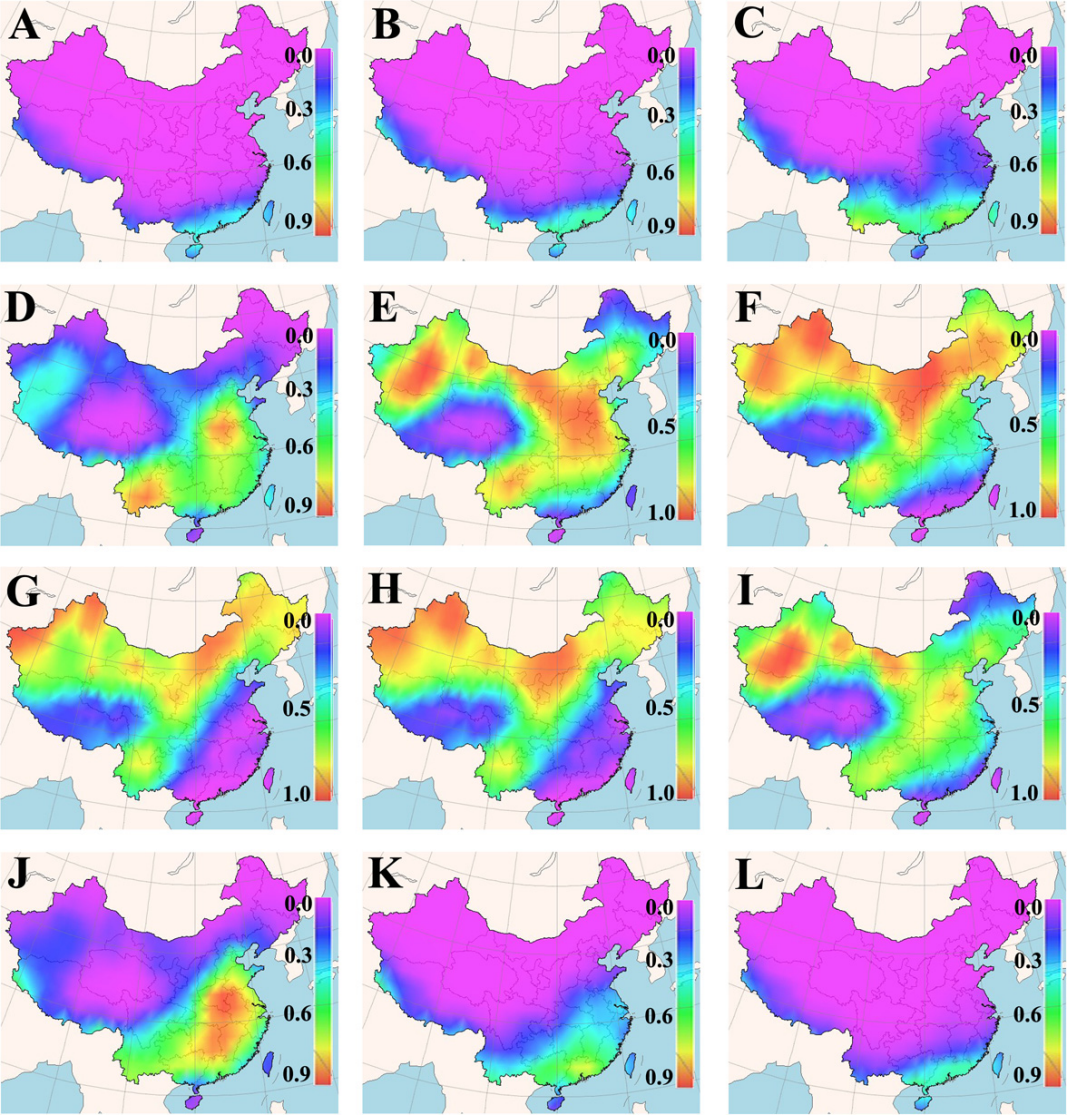
Maps of monthly germination suitability of ‘Diva’ in different regions of China; (A-L) January–December.

**Fig 3:**
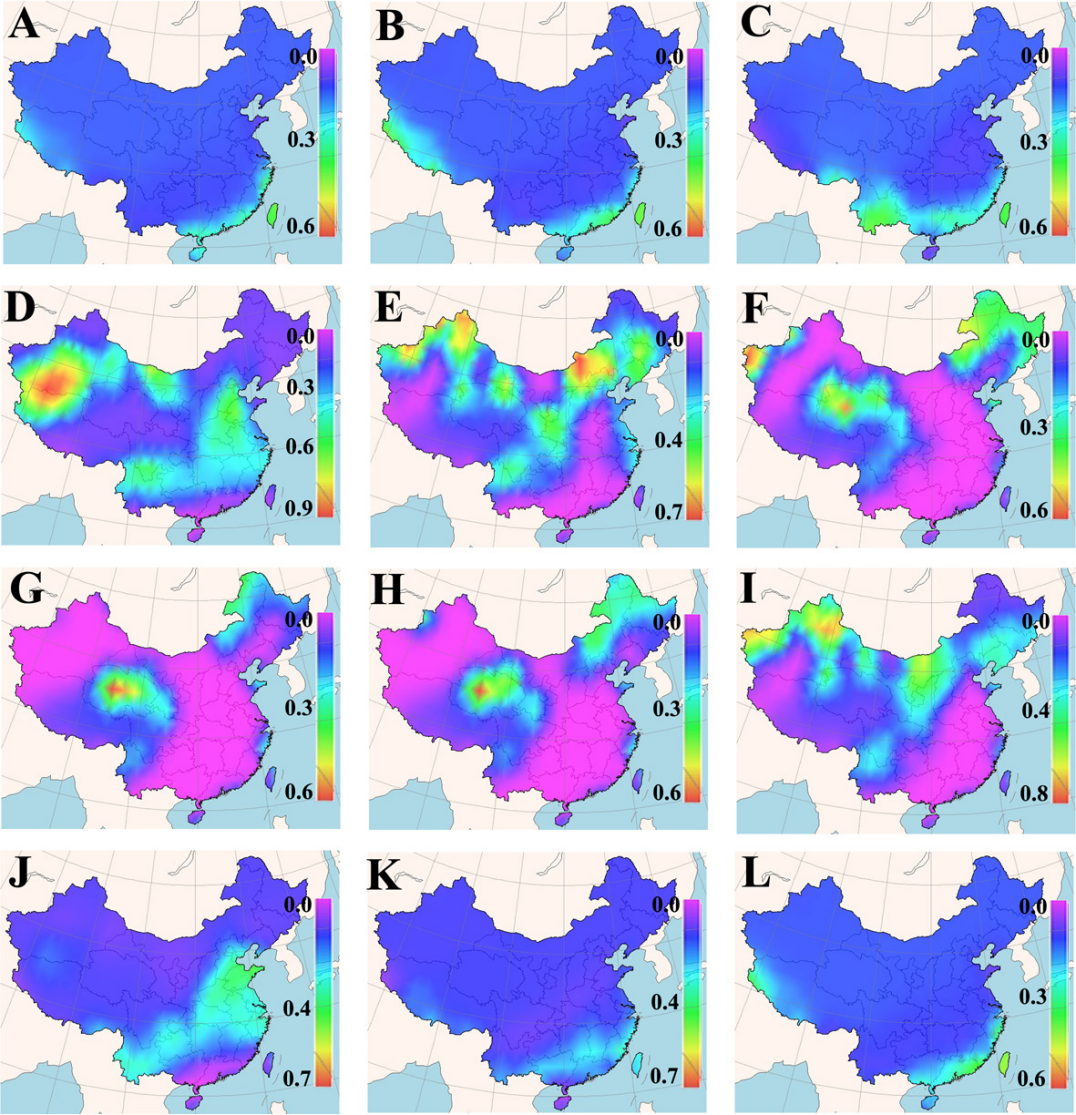
Maps of monthly germination suitability of ‘Rugby II’ in different regions of China; (A-L) January–December.

**Fig 4:**
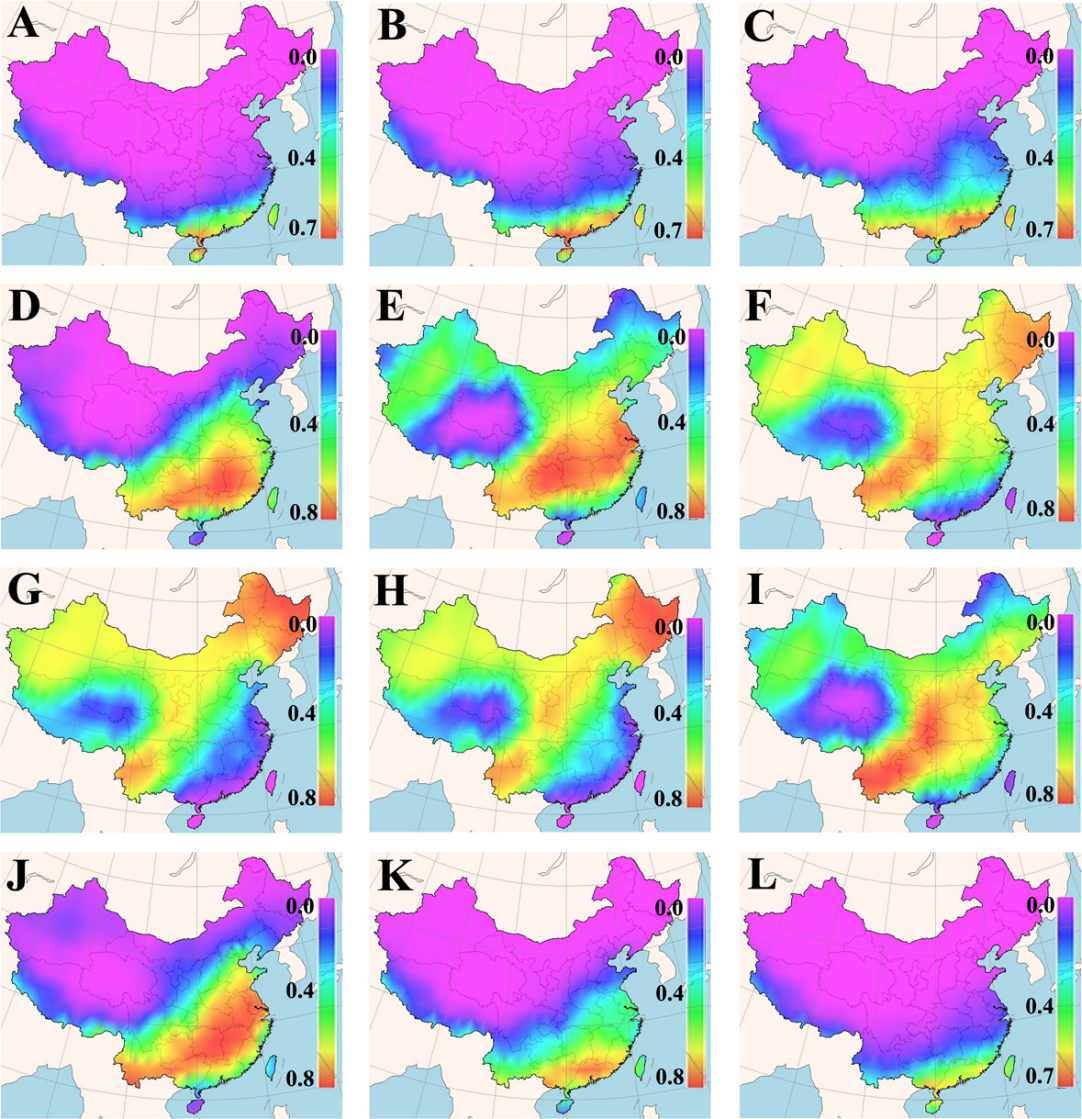
Maps of monthly germination suitability of ‘Leopard’ in different regions of China; (A-L) January–December.

**Fig 5:**
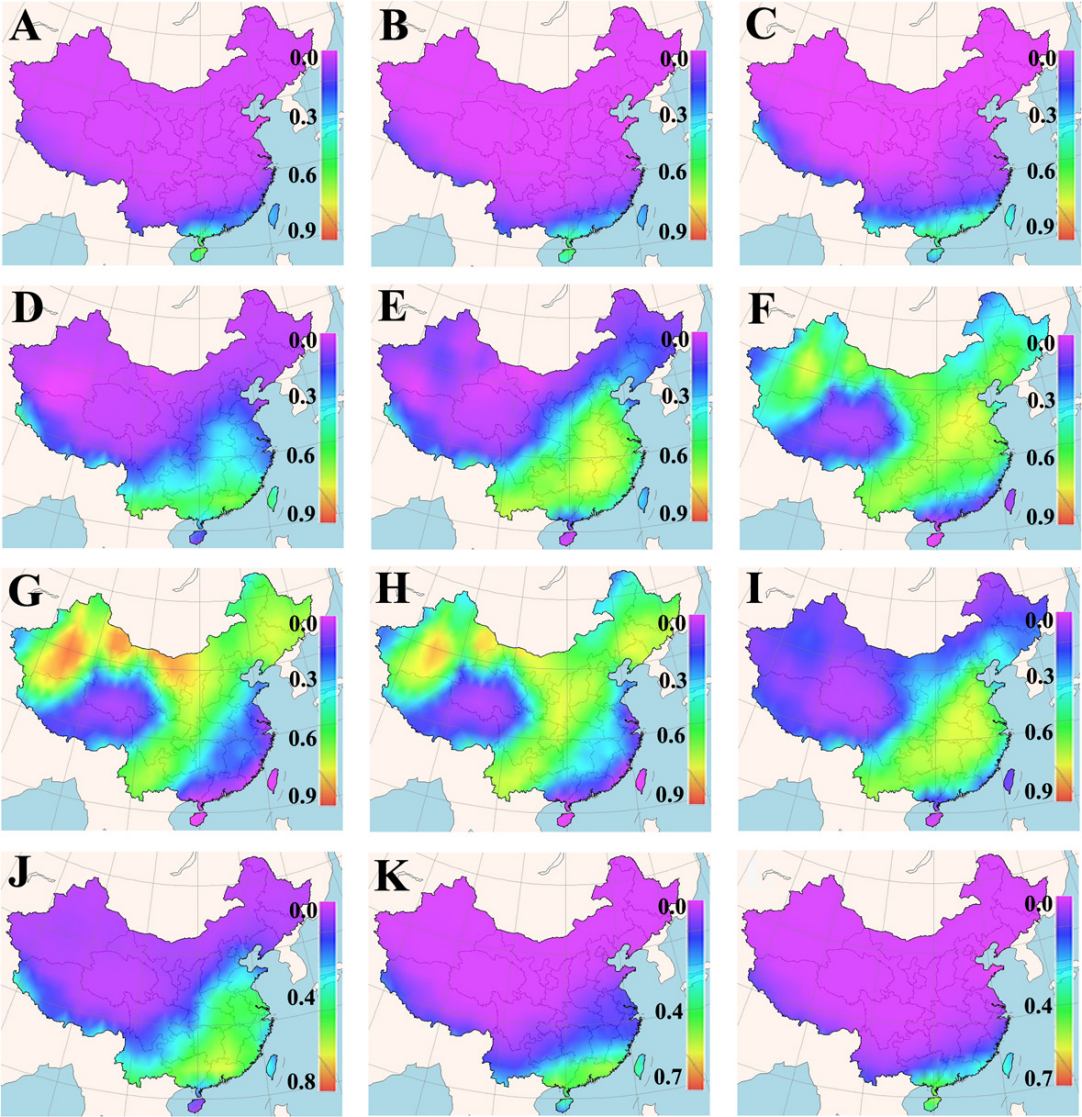
Maps of monthly germination suitability of ‘Sapphire’ in different regions of China; (A-L) January–December.

The cultivation suitability of a grass cultivar is defined by its acceptable germination percentage in the planned cultivation area for a particular period of time. Daily means of minimum and maximum earth surface temperature for a 25-years period in each cell of the Chinese map grid were fed into the new GA-BP-ANN temperature-germination functions, so as to predict a germination percentage for the tested cultivars in different months (Figures A~D in S1 File). The predicted germination percentages were subsequently converted to the suitability for the tested cultivars within each grid cell of the map via FreeMicaps (Figs 1–5). Among all the tested KB cultivars, suitability of ‘Rugby II’ was found to be the narrowest in both geological and time scales (Fig 3). In contrast, ‘Leopard’ was shown to have the widest suitability in both geological and time scales (Fig 4). To consider the germination capability, the sowing time of all five tested cultivars should not be arranged before March (Figs 1–5). However, the sowing time should not be later than October since the seedlings will face the cold stress in the later months.

Our results also showed that the fluctuation in temperature between day and night was an essential factor in facilitating seed germination of KB. The best evidence supporting this is that KB are documented to have very low germination in Hainan (the southernmost region in the map) and Taiwan (the southeast island in the map) provinces where the day/night temperatures are amenable around 20°C without substantial fluctuation from December to March [41]; all the five KB cultivars were also predicted to have very low germination in these two provinces during that period (Figs 1–5, Figures A~D in S1 File).

## Discussions

The mathematic models to correlate environmental factors and germination responses of grasses have significantly contributed to the selection of suitable grasses for various regions with different environmental conditions [42–44]. These prediction models could provide efficient approaches to evaluate desirable characteristics and even to identify new traits of a candidate grass species that help it to prevail in new environment conditions [45]. These models could also help elucidating the relationship between genotype and germination-related phenotypes to support rapid expansion in the cultivation of various grass cultivars [45]. Furthermore, these environmental factor-germination response models could also provide us data to foretell how the changes in agricultural systems could influence the grass germination [10,46]. Grass scientists have already developed various models for simulating the seed germination responses to temperature conditions [10,42–44]. This research tries to construct simulating models based on the unsupervised GA-BP-ANN, which automatically generates regressions directly from the inputted experimental data. When combined with a visual suitability map, these new regression models could provide decision makers a confident approach to select grass species and to plan seeding times [11,12].

The back propagation (BP) network is the most widely used for nonlinear relationship simulation [47]. The BP based simulation belongs to supervised learning; its training process has two phases: forward propagation and backward propagation [48]. In the forward propagation, the weighted and threshold values of each layer are calculated by iteration and passed into the three-layer BP network. The backward propagation (BP) uses the weighted and threshold values for revision [49]. In this study, the BP algorithm was used to smoothing the surface of nonlinear temperature-germination responses and it was proven to effectively optimize both core functions of quintic equation and GA.

The quadratic response surface used to be a dominant method for analyzing grass seed germination performance under a series of temperature regimes, especially to test the impact of diurnal temperature treatments on the seed germination [11,12]. However, the two-dimensional response surface could not show the global fitting error between the quadratic function and the experimental data [10,12]; the drawback of high prediction errors (RMSE ranges from 0.21 to 0.23) could not be ignored. Consistent with our previous study [10], the quadratic/quintic equation showed significantly lower fitting errors and higher confidences than their corresponding general quadratic/quintic ones (Table 7). That might be because the temperature-germination correlation was nonlinear [10]. Significantly, the GA-BP-ANN models for all tested five cultivars demonstrate lowest prediction errors (RMSE ranges from 0.02 to 0.09). Moreover, the GA-BP-ANN models provide with us a more reasonable prediction on region/season suitability, especially in the warm regions with less temperature fluctuation between day and night (eg. Hainan and Taiwan). The tested germination percentages in warm regimes (Table 1–5) are very low when the temperature decreases are less than 5°C from T_1_ to T_2_.

We introduced a temperature-germination response model, the GA-BP-ANN, in this study for predicting optimal sowing region and timing of five KB cultivars. It shows better performances than several published models, including general quadratic regression and BP-ANN based quadratic/quintic equations in two main aspects. Firstly, the construction of temperature-germination response is simplified since there is no requirement on selections of core functions (such as quintic equation in previous study [10]) and parameters for fitting the proposed GA-BP-ANN models. Secondly, the GA-BP-ANN models showed a better fit (lower RMSE values) than BP-ANN models previously developed. However, the present version of GA-BP-ANN model for germination response still has potential for further improvement in several aspects. For example, data of field experiences for seed temperature-germination response should be collected for further validating these GA-BP-ANN models of KB cultivars. As suggested by Hardegree and Van Vactor, both field-variable and chamber-variable temperature-germination response data should be included in the regression equations [50–53]. Moreover, the advantage of GA-BP-ANN should be utilized in building plant response prediction module to other environmental factors which also influence the seed germination. Bradford [54] quantified the seed germination behaviors upon a wide array of environmental conditions, such as temperature, water potential, so as to build general germination-response models of grass seed. In addition, plant responses to environmental factors at different growth stages, especially the seedling stage, should be tested in laboratory conditions in future research, and the results could be applied to building GA-BP-ANN models to predict the growth performance of crop in field. GA-BP-ANN model predictions of crop responses to different environmental factors at various developing stage would provide more, reliable information for us to select optimal planting regions and sowing times for various grasses and crops.

### Conclusions

In this study, we tested the influence of diurnal fluctuations of temperatures on seed germination of five KB cultivars (‘Midnight II’, ‘Diva’, ‘Rugby II’, ‘Leopard’, ‘Sapphire’). Optimum germination for the five tested cultivars was observed at four fluctuating temperature regimes: 20/25, 15/30, 20/30, and 25/30°C. Germination percentages of all five cultivars were found to be lower than 50% at constant temperature regimes ranging from 20 to 30°C.

Both automatic GA-BP-ANN and other regressions were utilized to simulate the grass temperature-germination response function in the current study. Since the GA-BP-ANN method is independent of empirical assumptions, artificial bias is not to be a concern of its prediction. When used by different researchers, the GA-BP-ANN method should always produce unbiased results although the researchers might have different preferences in their selection of the core equation. Therefore, this GA-BP-ANN approach will provide us a user-friendly way to tackle the temperature-germination problem and achieve very high prediction accuracies.

Based on the experimentally derived GA-BP-ANN models and available climate data, a seed-suitability national map of China for the five KB cultivars was generated. The suitability of ‘Rugby II’ was found to fit the narrowest spatial and temporal ranges, while ‘Leopard’ fit the widest ranges (Fig 1). In addition, the seed sowing time of tested KB should be arranged from March to October.

## Material and Methods

### Seeds and conditioning

The widely used KB cultivars in China (‘Midnight II’, ‘Diva’, ‘Rugby II’, ‘Leopard’, and ‘Sapphire’) were tested in this study. The grass seeds were purchased from Shanghai Chunyin Turf Inc., (Shanghai, China) and stored at room temperature until use. Seed viability was evaluated on moistened filter paper at 25/25°C on receipt of the seeds and after the germination experiments [12]. Finally, the loss of viability during storage was found less than 1% [10,12].

Seeds were surface sterilized by soaking in 0.01% HgCl_2_ for one minute and rinsed four times with sterilized distilled water. Then the seeds were placed on moistened filter papers in petri dishes and grown in different incubators consisting of 36 different regimes of diurnal temperature fluctuations: briefly 16 hours of day time at temperature T_1_ and 8 hours of night time at temperature T_2_. Both T_1_ and T_2_ ranged from 5 to 40°C with 5°C increments [12]. Germination counts were conducted daily until no further germination occurred after about 15~20 days (S2 File). In each experiment, three replicas of 50 seeds were tested.

### Genetic algorithm

Genetic algorithms (GA) is an iterative stochastic optimization approach inspired by nature’s evolutionary genetics: the most fit individual has the highest chance of survival [55,56]. The GA method was widely used in solving many nonlinear optimization problems, including those found in computational biology [57–59]. In this study, the GA method is used to generate the fittest mapping function between the independent variable (the temperature matrix T) and dependent variable (germination percentage matrix G), where T = (t_1i,_ t_2i_), G = (g_i_), i = 1, 2, 3… M (M is the number of tested temperature regimes with replication), and g_i_ ranges in the interval [0, 1].

As mentioned above, the GA approach simulates the survival of the fittest individuals in the population, controlled by the definition of a fitness score [39]. An initial individual is a map function generated randomly to describe the relationship between the selected temperature matrix T' and its corresponding germination percentage matrix G', where T' = (t_1j,_ t_2j_), G' = (g_j_), and j < i. Different functions represent different solutions for the temperature-germination problem. In general, the genetic algorithm contains the following steps in a sequential order: initial population selection, fitness function evaluation, individuals selection, population reproduction, individuals’ crossover and mutation operation modules [55]. In the meantime, the back propagation artificial neural network was also integrated for solution optimization [10]. To construct the GA-BP-ANN model, the observed 108 pairs temperature-germination data in 36 regimes of each cultivar, were divided into a training set (90 observations, 83%) and a test set (18 observations, 17%). The training set was chosen to cover all temperature regimes from 5/5 to 40/40°C. Model validation was performed on 5/5, 40/40, 25/25°C, etc, (test sets) which represented severe and moderate temperature conditions for seed germination, respectively [39]. The Root Mean Square Errors (RMSE) was used to evaluate the fitness and predictive capability [60].

As a reference to the performance of GA-BP-ANN models, the previous published based regression methods, like general quadratic/quintic and BP optimized quadratic/quintic models (Figure E~I in S1 File, S3 File) were also utilized to simulate temperature-germination responses [61]. The generalized quadratic equation was [12]: Y_1_ = A_0_+ A_1_*T_1_+ A_2_*T_2_+ A_3_*T_1_^2^+ A_4_*T_2_^2^+ A_5_*T_1_*T_2_, where Y_1_: percent predicted germination, A_0_: intercept, A_1_ through A_5_: coefficients, T_1,_ and T_2_: diurnal fluctuations of temperature. The general quintic equation was previously described [10]. Briefly, it could be presented as Y1=∑m=15{∑n=0m[T1n*T2m-n]*f(A)}+A0', where Y_1_: percent predicted germination, A_0_': intercept, and f(A): coefficient function.

### Spatial mapping

The grass suitability was represented by the germination percentage. The grass suitability maps were created using the FreeMicaps software (http://bbs.121323.com/guojf/FreeMicaps20111001.rar). Similar to the Surfer software [62], FreeMicaps also uses point (station) data on the grid that is compatible with GIS. The temperature values of adjacent regions around the station were generated using Cressman interpolation method [63,64]. Means of minimum and maximum of daily earth surface temperature in the grids made of 313 weather stations (Figures A~D in S1 File) and for a period of 25-years (from 1983 to 2007) were calculated from the data sets obtained at the American National Aeronautics and Space Administration (NASA) website (http://power.larc.nasa.gov/cgi-bin/cgiwrap/solar/sse.cgi?grid@larc.nasa.gov#s11). The mean, minimum, and maximum daily temperatures over the grids were used as the T_2_ and T_1_ variables respectively in the GA-BP-ANN functions for calculating germination percentages (grass suitability).

## Supporting Information

S1 File Mean of day/night temperatures from January to March (Figure A), from April to June (Figure B), from July to September (Figure C) and from October to December (Figure D) in China; Regression performance of GA-BP ANN in simulating the temperature-germination response of ‘Midnight II’ (Figure E), ‘Diva’ (Figure F), ‘Rugby II’ (Figure G), ‘Leopard’ (Figure H) and ‘Sapphire’ (Figure I). (DOCX)

S2 File Raw data of seed germination. (XLS)

S3 File Regression equations. (DOCX)
