# Supplementary material for: Application of Genetic Algorithm to Predict Optimal Sowing Region and Timing for Kentucky Bluegrass in China
Source: PLoS One. 2015 Jul 8;10(7):e0131489. doi: 10.1371/journal.pone.0131489 (PMC4496032; doi:10.1371/journal.pone.0131489)
Supplement: S1 File — (DOCX) [file pone.0131489.s001.docx]

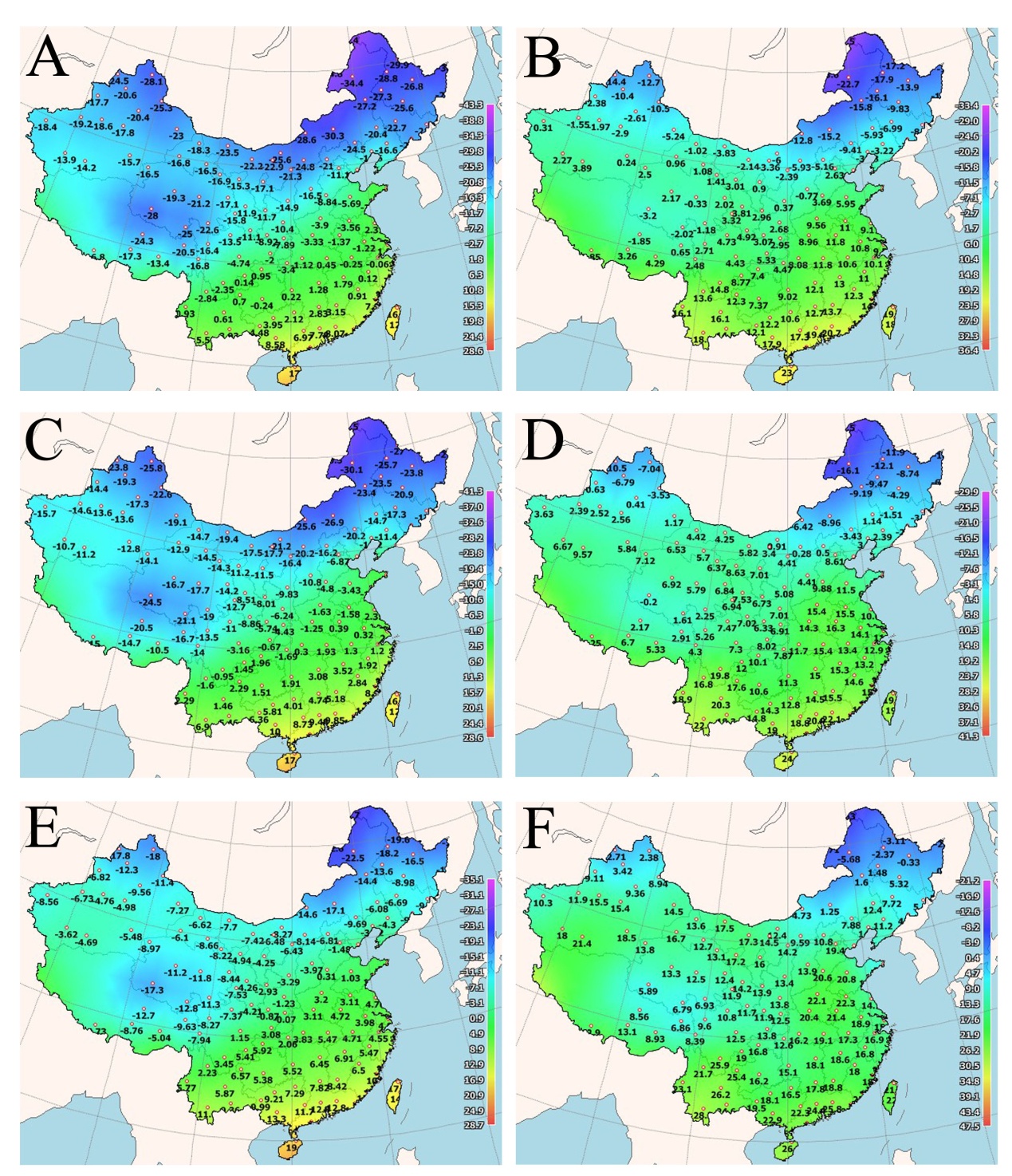


**S1 Fig. A. Mean of day/night temperatures in January, February and March in China. A, C, E: means of night temperature in January, February and March; B, D, F: means of night temperature in January, February and March.**


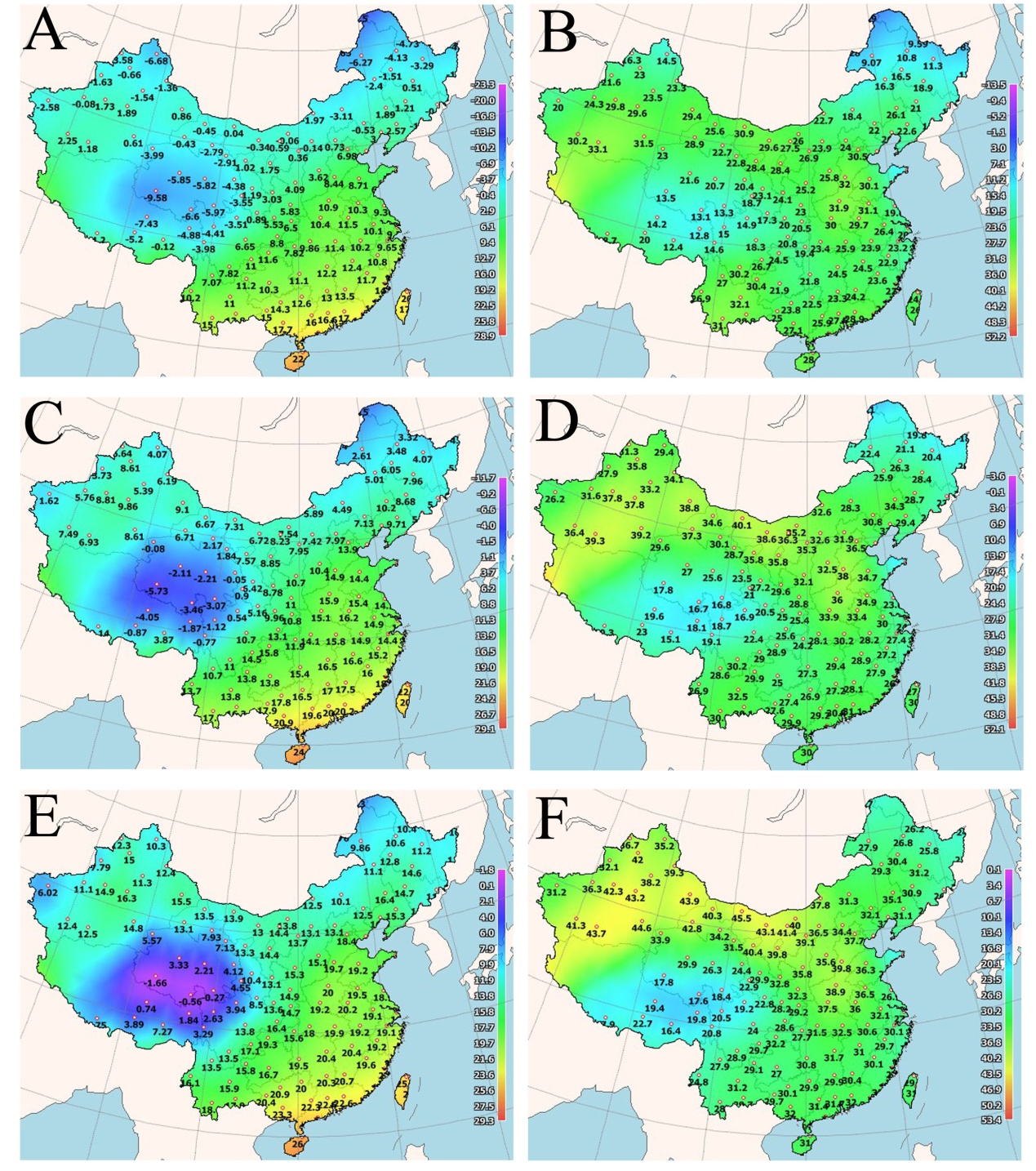


**S1 Fig. B. Means of day/night temperatures in April, May and June in China. A, C, E: means of night temperature in April, May and June; B, D, F: means of night temperature in April, May and June.**


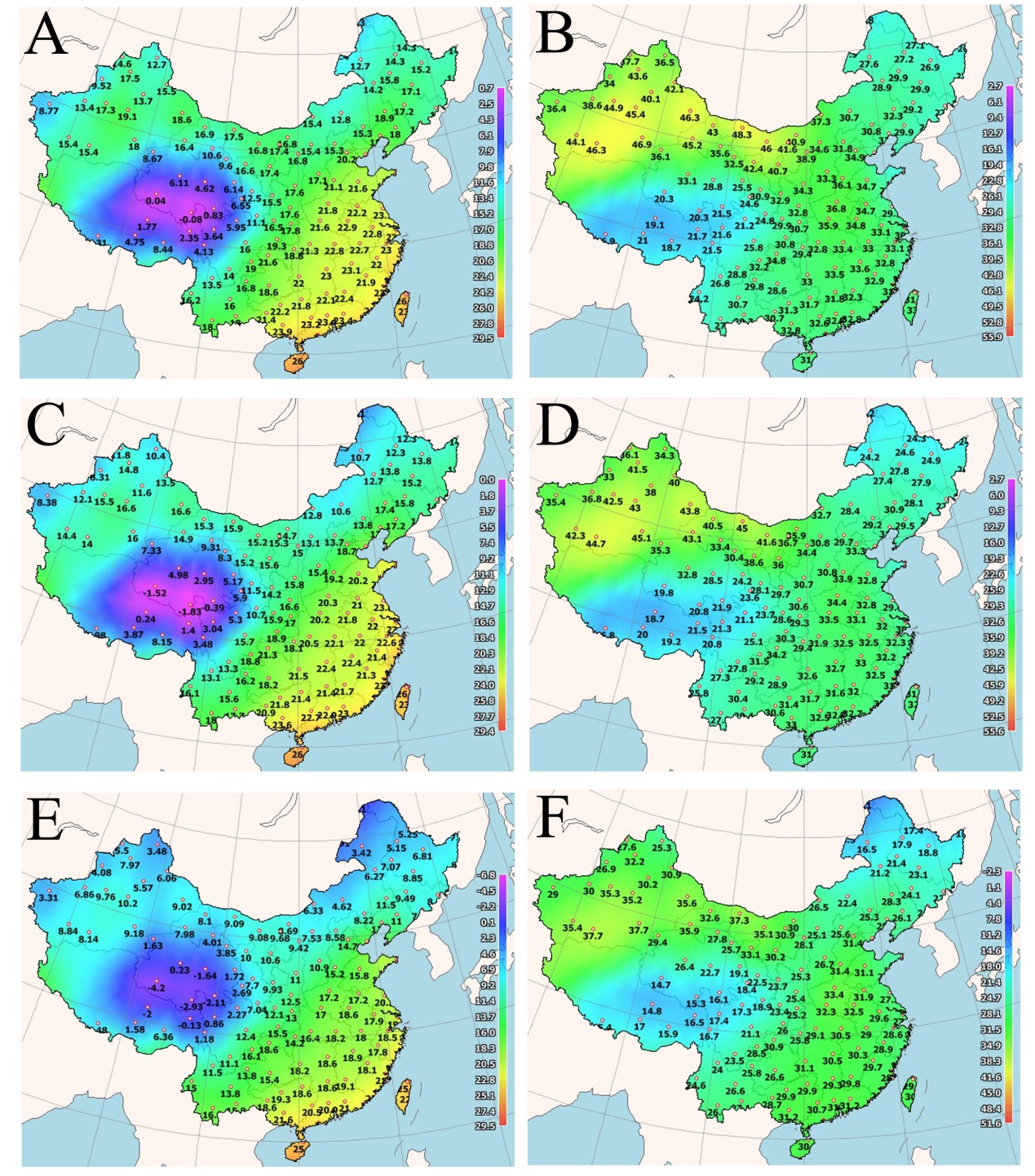


**S1 Fig. C. Means of day/night temperatures in July, August and September in China. A, C, E: means of night temperature in sequential July, August and September; B, D, F: means of night temperature in July, August and September.**


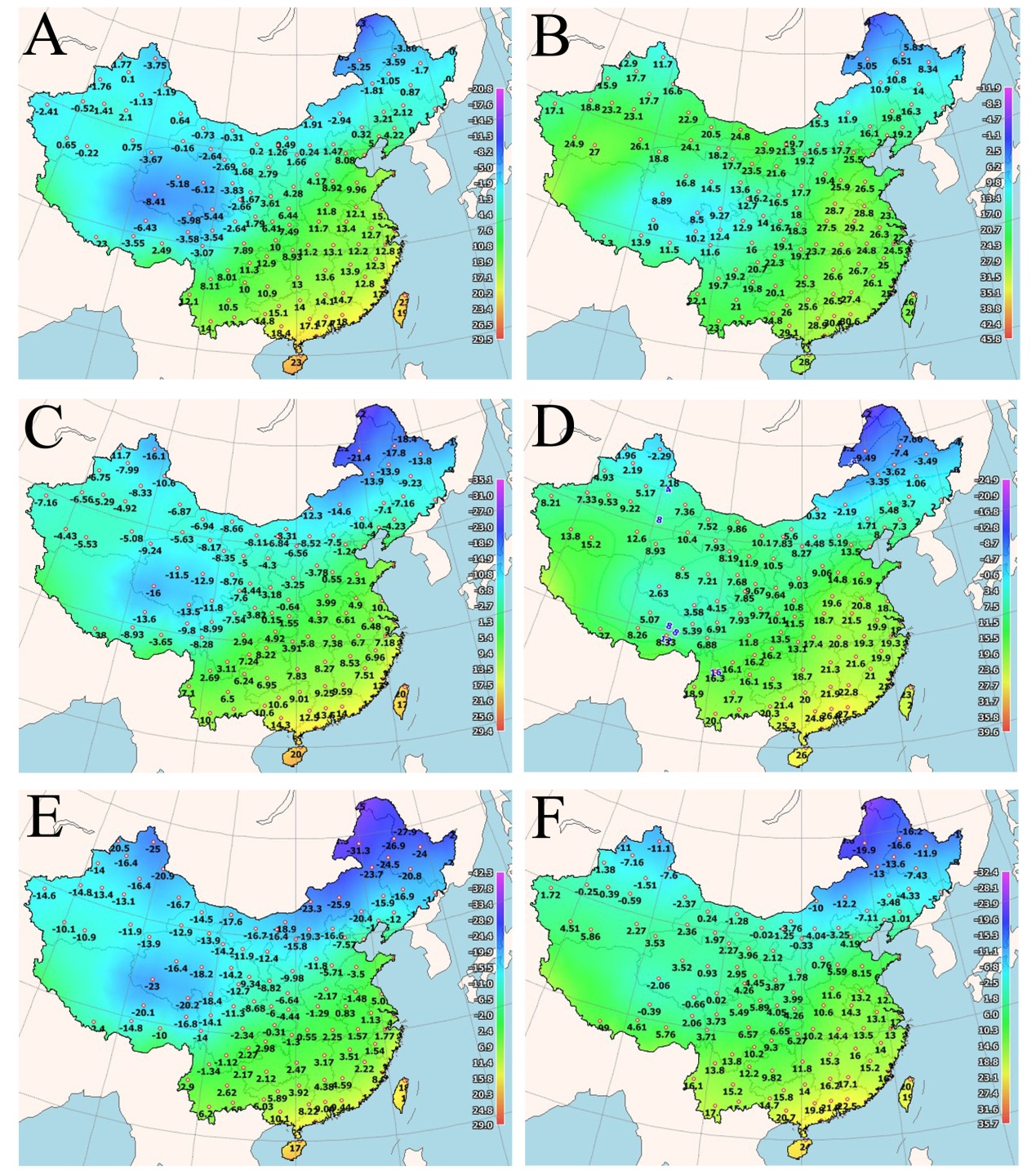


**S1 Fig. D. Means of day/night temperatures in October, November and December in China. A, C, E: means of night temperature in October, November and December; B, D, F: means of night temperature in October, November and December.**


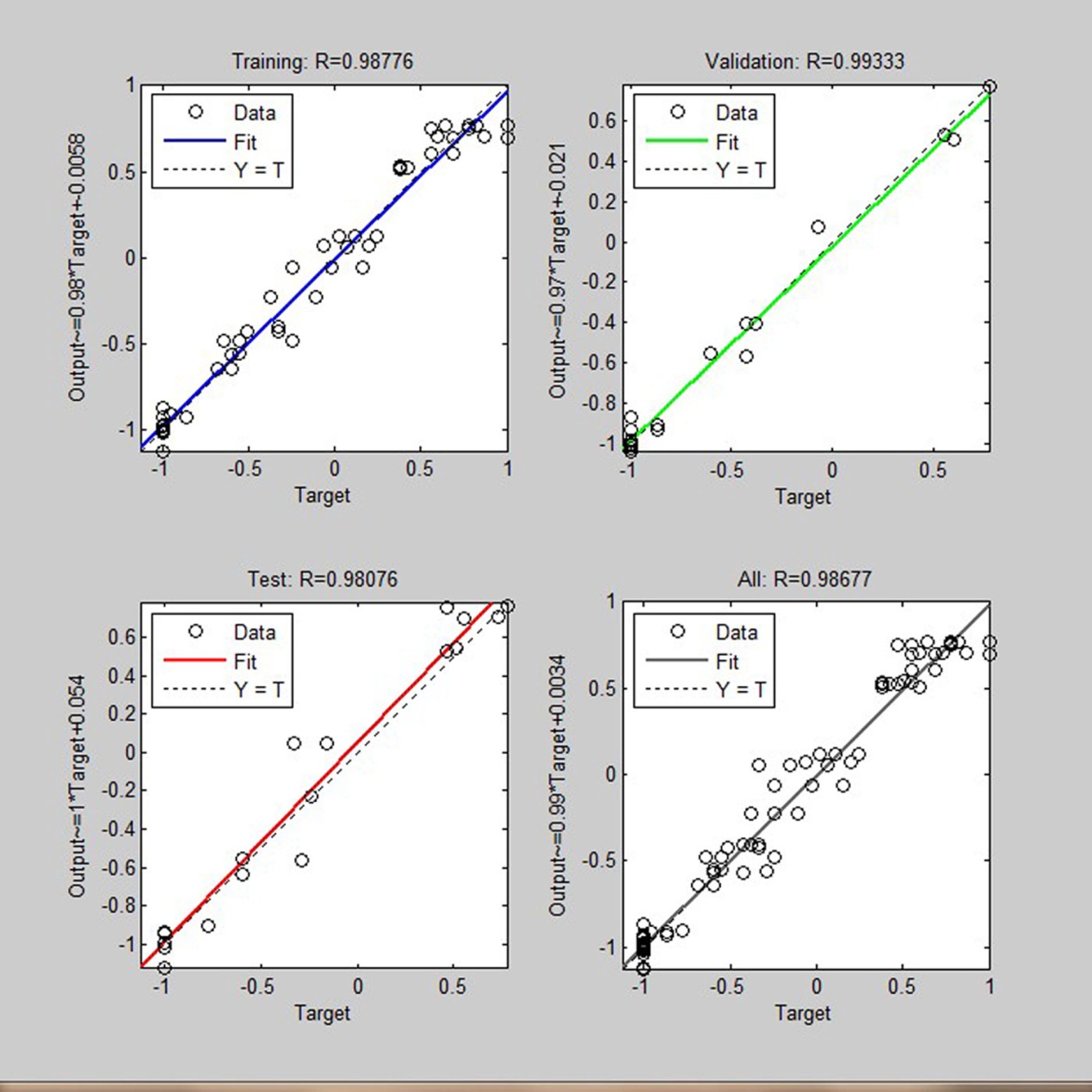


**S1 Fig. E. Regression performance of GA-BP ANN in simulating the temperature-germination response of ‘Midnight II’.**


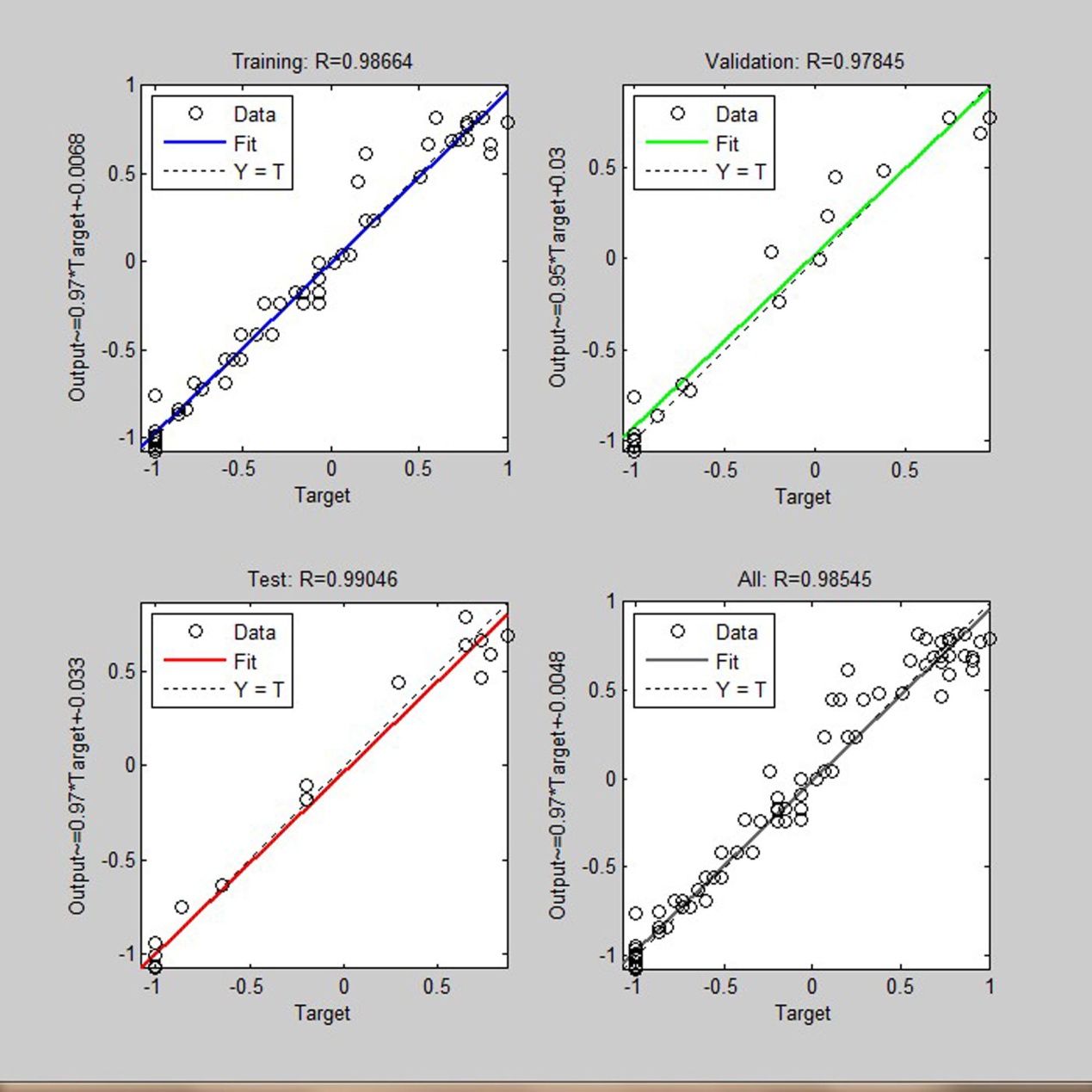


**S1 Fig. F. Regression performance of GA-BP ANN in simulating the temperature-germination response of ‘Diva’.**


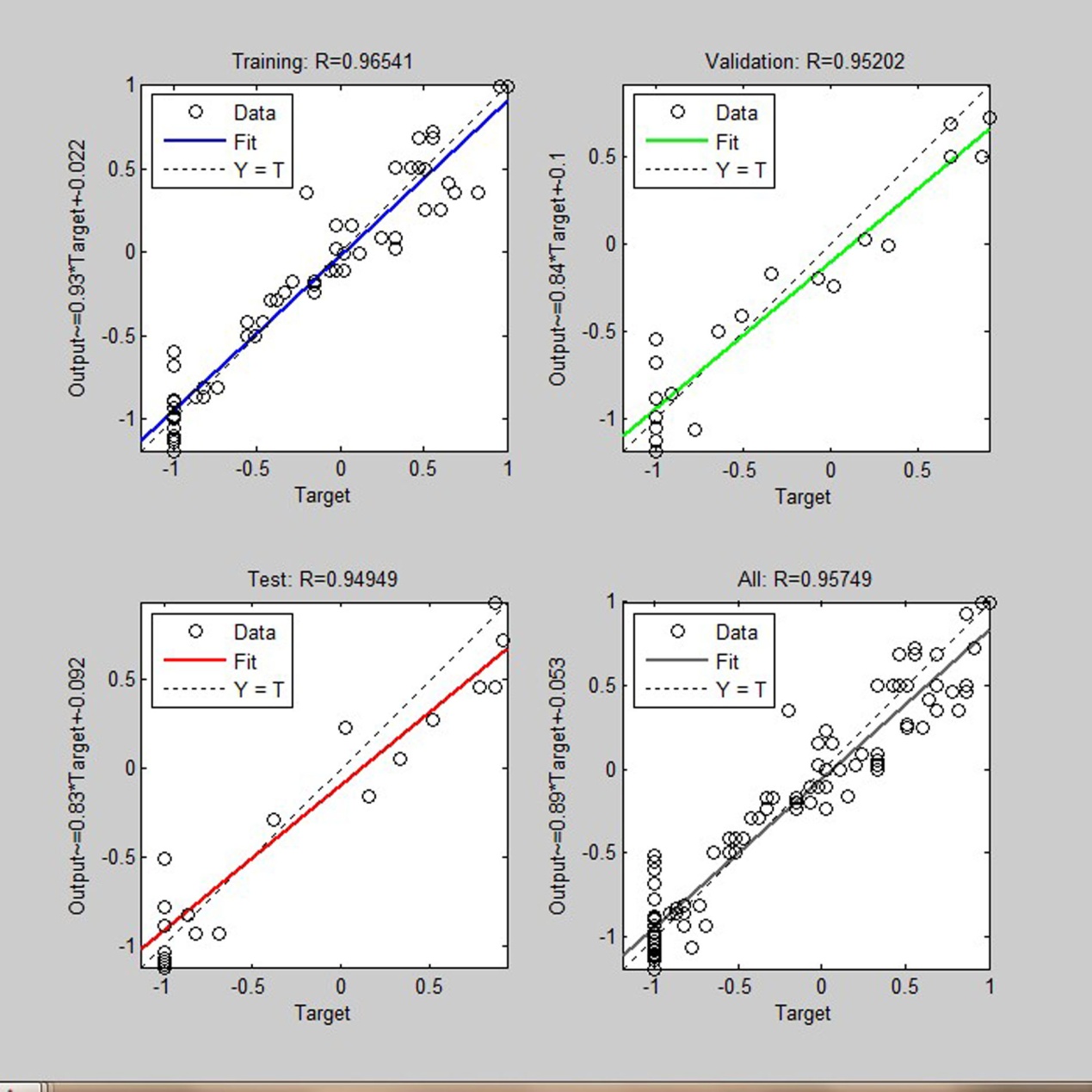


**S1 Fig. G. Regression performance of GA-BP ANN in simulating the temperature-germination response of ‘Rugby II’.**


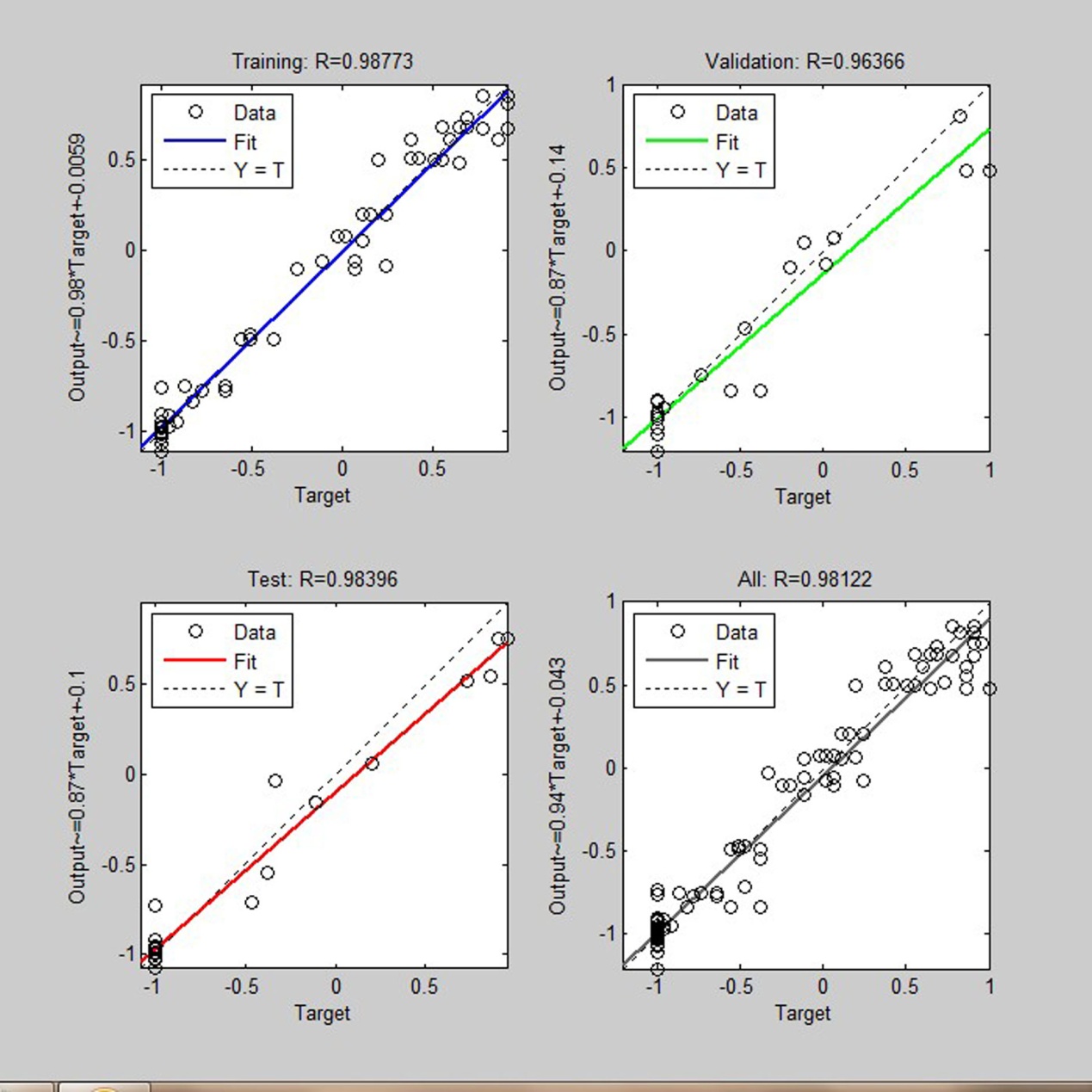


**S1 Fig. H. Regression performance of GA-BP ANN in simulating the temperature-germination response of ‘Leopard’.**


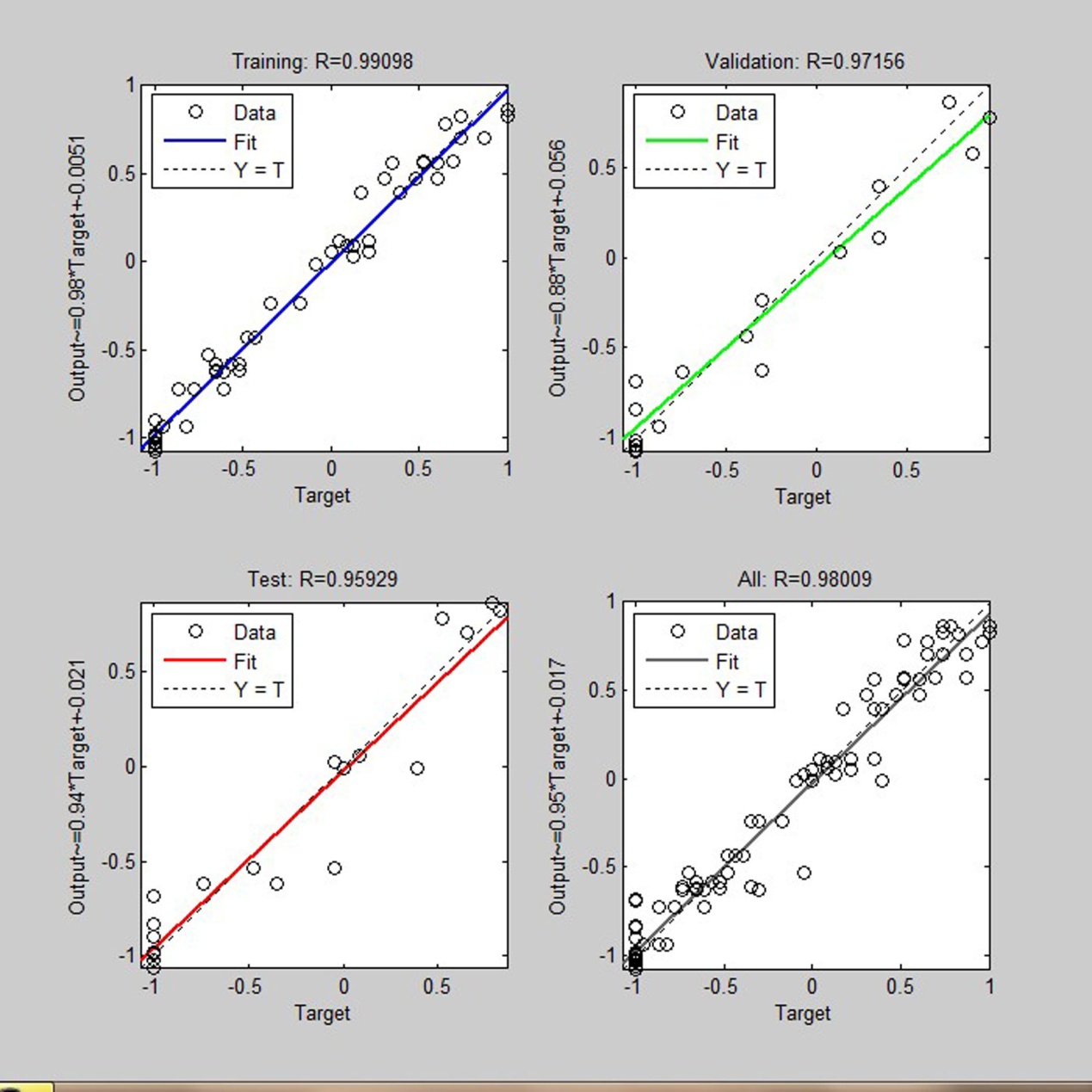


**S1 Fig. I. Regression performance of GA-BP ANN in simulating the temperature-germination response of ‘Sapphire’.**
