# Supplementary material for: Application of Genetic Algorithm to Predict Optimal Sowing Region and Timing for Kentucky Bluegrass in China
Source: PLoS One. 2015 Jul 8;10(7):e0131489. doi: 10.1371/journal.pone.0131489 (PMC4496032; doi:10.1371/journal.pone.0131489)
Supplement: S3 File — (DOCX) [file pone.0131489.s003.docx]

Appendix B. Supplementary material

List of nonlinear equations generated for five cultivars

General quadratic equation for **cultivar ‘Midnight II’**:

Linear model Poly22:

f(x,y) = p00 + p10*x + p01*y + p20*x^2 + p11*x*y + p02*y^2

Coefficients (with 95% confidence bounds):

p00 = -0.8581 (-1.327, -0.389)

p10 = 0.09911 (0.05648, 0.1417)

p01 = 0.01593 (-0.02837, 0.06023)

p20 = -0.002231 (-0.003215, -0.001247)

p11 = 0.001096 (-0.0005119, 0.002703)

p02 = -0.001316 (-0.0023, -0.0003316)

BP-ANN-Quadratic equation for **cultivar ‘Midnight II’:**

Linear model Poly22:

f(x,y) = p00 + p10*x + p01*y + p20*x^2 + p11*x*y + p02*y^2

Coefficients (with 95% confidence bounds):

p00 = -0.8533 (-1.316, -0.3903)

p10 = 3.974 (2.291, 5.657)

p01 = 0.5861 (-1.163, 2.335)

p20 = -3.607 (-5.162, -2.053)

p11 = 1.903 (-0.636, 4.442)

p02 = -2.182 (-3.737, -0.6272)

General quintic equation for **cultivar ‘Midnight II’:**

Linear model Poly55:

f(x,y) = p00 + p10*x + p01*y + p20*x^2 + p11*x*y + p02*y^2 + p30*x^3 + p21*x^2*y

+ p12*x*y^2 + p03*y^3 + p40*x^4 + p31*x^3*y + p22*x^2*y^2

+ p13*x*y^3 + p04*y^4 + p50*x^5 + p41*x^4*y + p32*x^3*y^2

+ p23*x^2*y^3 + p14*x*y^4 + p05*y^5

Coefficients (with 95% confidence bounds):

p00 = -0.7419 (-3.037, 1.553)

p10 = 0.5247 (-0.02075, 1.07)

p01 = -0.263 (-0.9777, 0.4516)

p20 = -0.09445 (-0.1631, -0.02584)

p11 = 0.07456 (-0.03631, 0.1854)

p02 = -0.01141 (-0.09551, 0.07268)

p30 = 0.006438 (0.002418, 0.01046)

p21 = -0.006581 (-0.01558, 0.002416)

p12 = 0.002822 (-0.006441, 0.01209)

p03 = -0.0008857 (-0.005432, 0.003661)

p40 = -0.0001795 (-0.0002866, -7.232e-005)

p31 = 0.0002 (-0.0001171, 0.000517)

p22 = -6.026e-005 (-0.0005123, 0.0003918)

p13 = -3.272e-005 (-0.000356, 0.0002905)

p04 = 2.583e-005 (-8.759e-005, 0.0001393)

p50 = 1.74e-006 (6.878e-007, 2.793e-006)

p41 = -1.93e-006 (-5.852e-006, 1.992e-006)

p32 = -2.012e-007 (-7.464e-006, 7.061e-006)

p23 = 1.704e-006 (-5.559e-006, 8.966e-006)

p14 = -1.023e-006 (-4.945e-006, 2.9e-006)

p05 = 1.504e-007 (-9.02e-007, 1.203e-006)

BP-ANN-Quintic equation for **cultivar ‘Midnight II’:**

Linear model Poly55:

f(x,y) = p00 + p10*x + p01*y + p20*x^2 + p11*x*y + p02*y^2 + p30*x^3 + p21*x^2*y

+ p12*x*y^2 + p03*y^3 + p40*x^4 + p31*x^3*y + p22*x^2*y^2

+ p13*x*y^3 + p04*y^4 + p50*x^5 + p41*x^4*y + p32*x^3*y^2

+ p23*x^2*y^3 + p14*x*y^4 + p05*y^5

Coefficients (with 95% confidence bounds):

p00 = 0.5893 (-1.137, 2.316)

p10 = 4.871 (-11.54, 21.28)

p01 = -14.92 (-36.43, 6.584)

p20 = -117.4 (-200, -34.82)

p11 = 261.6 (128.2, 395.1)

p02 = -88.33 (-189.5, 12.89)

p30 = 401.2 (207.6, 594.7)

p21 = -822.8 (-1256, -389.6)

p12 = 217.2 (-228.8, 663.2)

p03 = 79.04 (-139.8, 297.9)

p40 = -481.9 (-688.3, -275.6)

p31 = 910.6 (299.9, 1521)

p22 = -16.2 (-886.7, 854.3)

p13 = -362.6 (-985.2, 259.9)

p04 = 79.57 (-138.9, 298)

p50 = 192.7 (111.6, 273.7)

p41 = -334.4 (-636.5, -32.24)

p32 = -113 (-672.5, 446.4)

p23 = 284.2 (-275.3, 843.7)

p14 = -80.06 (-382.2, 222.1)

p05 = 0.1514 (-80.92, 81.23)

General quadratic equation for **cultivar ‘Diva’**:

Linear model Poly22:

f(x,y) = p00 + p10*x + p01*y + p20*x^2 + p11*x*y + p02*y^2

Coefficients (with 95% confidence bounds):

p00 = -0.9023 (-1.376, -0.429)

p10 = 0.1053 (0.0623, 0.1483)

p01 = 0.01298 (-0.03171, 0.05768)

p20 = -0.00238 (-0.003373, -0.001387)

p11 = 0.001264 (-0.000358, 0.002886)

p02 = -0.001392 (-0.002385, -0.0003983)

BP-ANN-quadratic equation for **cultivar ‘Diva’**:

Linear model Poly22:

f(x,y) = p00 + p10*x + p01*y + p20*x^2 + p11*x*y + p02*y^2

Coefficients (with 95% confidence bounds):

p00 = -0.9047 (-1.366, -0.4433)

p10 = 4.241 (2.564, 5.918)

p01 = 0.4923 (-1.25, 2.235)

p20 = -3.842 (-5.391, -2.293)

p11 = 2.086 (-0.4439, 4.615)

p02 = -2.263 (-3.812, -0.7135)

General quintic equation for **cultivar ‘Diva’**:

Linear model Poly55:

f(x,y) = p00 + p10*x + p01*y + p20*x^2 + p11*x*y + p02*y^2 + p30*x^3 + p21*x^2*y

+ p12*x*y^2 + p03*y^3 + p40*x^4 + p31*x^3*y + p22*x^2*y^2

+ p13*x*y^3 + p04*y^4 + p50*x^5 + p41*x^4*y + p32*x^3*y^2

+ p23*x^2*y^3 + p14*x*y^4 + p05*y^5

Coefficients (with 95% confidence bounds):

p00 = -0.741 (-2.606, 1.124)

p10 = 0.4714 (0.02804, 0.9147)

p01 = -0.1892 (-0.7701, 0.3917)

p20 = -0.08895 (-0.1447, -0.03319)

p11 = 0.07204 (-0.01808, 0.1622)

p02 = -0.0198 (-0.08815, 0.04855)

p30 = 0.006036 (0.002769, 0.009304)

p21 = -0.005571 (-0.01288, 0.001742)

p12 = 0.001694 (-0.005835, 0.009224)

p03 = 2.397e-005 (-0.003671, 0.003719)

p40 = -0.0001661 (-0.0002532, -7.904e-005)

p31 = 0.0001512 (-0.0001065, 0.000409)

p22 = -8.791e-008 (-0.0003675, 0.0003673)

p13 = -5.945e-005 (-0.0003222, 0.0002033)

p04 = 1.692e-005 (-7.527e-005, 0.0001091)

p50 = 1.598e-006 (7.428e-007, 2.454e-006)

p41 = -1.417e-006 (-4.605e-006, 1.771e-006)

p32 = -4.513e-007 (-6.354e-006, 5.452e-006)

p23 = 8.967e-007 (-5.006e-006, 6.8e-006)

p14 = 8.303e-008 (-3.105e-006, 3.271e-006)

p05 = -1.661e-007 (-1.021e-006, 6.894e-007)

BP-ANN-Quintic equation for **cultivar ‘Diva’**:

Linear model Poly55:

f(x,y) = p00 + p10*x + p01*y + p20*x^2 + p11*x*y + p02*y^2 + p30*x^3 + p21*x^2*y

+ p12*x*y^2 + p03*y^3 + p40*x^4 + p31*x^3*y + p22*x^2*y^2

+ p13*x*y^3 + p04*y^4 + p50*x^5 + p41*x^4*y + p32*x^3*y^2

+ p23*x^2*y^3 + p14*x*y^4 + p05*y^5

Coefficients (with 95% confidence bounds):

p00 = 0.7407 (-0.7038, 2.185)

p10 = 15.42 (1.682, 29.15)

p01 = -26.07 (-44.07, -8.078)

p20 = -136.4 (-205.5, -67.32)

p11 = 150.1 (38.39, 261.7)

p02 = 35.41 (-49.28, 120.1)

p30 = 386.9 (225, 548.8)

p21 = -428.1 (-790.6, -65.67)

p12 = 59.74 (-313.4, 432.9)

p03 = -110.3 (-293.5, 72.8)

p40 = -440.1 (-612.8, -267.5)

p31 = 499.5 (-11.44, 1010)

p22 = -56.11 (-784.5, 672.3)

p13 = -29.39 (-550.3, 491.5)

p04 = 103.8 (-78.96, 286.6)

p50 = 173.9 (106.1, 241.8)

p41 = -201.7 (-454.5, 51.11)

p32 = -9.084 (-477.2, 459)

p23 = 79.61 (-388.5, 547.7)

p14 = -49.56 (-302.4, 203.3)

p05 = -18.25 (-86.08, 49.59)

General quadratic equation for **cultivar ‘Rugby II’**:

Linear model Poly22:

f(x,y) = p00 + p10*x + p01*y + p20*x^2 + p11*x*y + p02*y^2

Coefficients (with 95% confidence bounds):

p00 = -0.876 (-1.339, -0.4132)

p10 = 0.1023 (0.06023, 0.1443)

p01 = 0.01319 (-0.03051, 0.05689)

p20 = -0.002301 (-0.003272, -0.00133)

p11 = 0.001197 (-0.0003887, 0.002783)

p02 = -0.001337 (-0.002309, -0.0003662)

BP-ANN-quadratic equation for **cultivar ‘Rugby II’**:

Linear model Poly22:

f(x,y) = p00 + p10*x + p01*y + p20*x^2 + p11*x*y + p02*y^2

Coefficients (with 95% confidence bounds):

p00 = -0.8724 (-1.318, -0.427)

p10 = 4.076 (2.457, 5.695)

p01 = 0.5314 (-1.151, 2.214)

p20 = -3.672 (-5.167, -2.176)

p11 = 1.931 (-0.5115, 4.373)

p02 = -2.162 (-3.657, -0.6662)

General quintic equation for **cultivar ‘Rugby II’**:

Linear model Poly55:

f(x,y) = p00 + p10*x + p01*y + p20*x^2 + p11*x*y + p02*y^2 + p30*x^3 + p21*x^2*y

+ p12*x*y^2 + p03*y^3 + p40*x^4 + p31*x^3*y + p22*x^2*y^2

+ p13*x*y^3 + p04*y^4 + p50*x^5 + p41*x^4*y + p32*x^3*y^2

+ p23*x^2*y^3 + p14*x*y^4 + p05*y^5

Coefficients (with 95% confidence bounds):

p00 = -0.1701 (-2.338, 1.997)

p10 = 0.3557 (-0.1594, 0.8709)

p01 = -0.3011 (-0.9761, 0.374)

p20 = -0.07908 (-0.1439, -0.01429)

p11 = 0.09139 (-0.01333, 0.1961)

p02 = -0.01907 (-0.0985, 0.06036)

p30 = 0.005749 (0.001952, 0.009545)

p21 = -0.007463 (-0.01596, 0.001035)

p12 = 0.002467 (-0.006283, 0.01122)

p03 = -0.0002367 (-0.004531, 0.004057)

p40 = -0.0001634 (-0.0002646, -6.219e-005)

p31 = 0.0002089 (-9.059e-005, 0.0005084)

p22 = -1.065e-005 (-0.0004376, 0.0004163)

p13 = -8.201e-005 (-0.0003873, 0.0002233)

p04 = 3.072e-005 (-7.641e-005, 0.0001379)

p50 = 1.598e-006 (6.036e-007, 2.592e-006)

p41 = -1.977e-006 (-5.682e-006, 1.728e-006)

p32 = -4.611e-007 (-7.321e-006, 6.399e-006)

p23 = 1.127e-006 (-5.732e-006, 7.987e-006)

p14 = 2.364e-007 (-3.468e-006, 3.941e-006)

p05 = -3.489e-007 (-1.343e-006, 6.451e-007)

BP-ANN-Quintic equation for **cultivar ‘Rugby II’**:

Linear model Poly55:

f(x,y) = p00 + p10*x + p01*y + p20*x^2 + p11*x*y + p02*y^2 + p30*x^3 + p21*x^2*y

+ p12*x*y^2 + p03*y^3 + p40*x^4 + p31*x^3*y + p22*x^2*y^2

+ p13*x*y^3 + p04*y^4 + p50*x^5 + p41*x^4*y + p32*x^3*y^2

+ p23*x^2*y^3 + p14*x*y^4 + p05*y^5

Coefficients (with 95% confidence bounds):

p00 = -0.7107 (-2.828, 1.407)

p10 = 8.016 (-12.12, 28.15)

p01 = 1.881 (-24.5, 28.26)

p20 = -52.26 (-153.5, 49.02)

p11 = 2.54 (-161.1, 166.2)

p02 = 5.998 (-118.2, 130.1)

p30 = 159.3 (-78.09, 396.7)

p21 = -112.6 (-643.9, 418.7)

p12 = 122.7 (-424.4, 669.7)

p03 = -76.06 (-344.5, 192.4)

p40 = -193.1 (-446.2, 59.99)

p31 = 192.9 (-556.1, 941.9)

p22 = -108.4 (-1176, 959.3)

p13 = -68.11 (-831.7, 695.5)

p04 = 84.6 (-183.3, 352.5)

p50 = 78.93 (-20.51, 178.4)

p41 = -87.31 (-457.9, 283.3)

p32 = -10.47 (-696.7, 675.7)

p23 = 129 (-557.2, 815.2)

p14 = -73.71 (-444.3, 296.9)

p05 = -3.069 (-102.5, 96.37)

General quadratic equation for **cultivar ‘Leopard’**:

Linear model Poly22:

f(x,y) = p00 + p10*x + p01*y + p20*x^2 + p11*x*y + p02*y^2

Coefficients (with 95% confidence bounds):

p00 = -0.8525 (-1.354, -0.3512)

p10 = 0.1075 (0.06193, 0.153)

p01 = 0.004143 (-0.0432, 0.05148)

p20 = -0.002504 (-0.003556, -0.001452)

p11 = 0.00171 (-8.243e-006, 0.003428)

p02 = -0.001607 (-0.002659, -0.0005546)

BP-ANN-quadratic equation for **cultivar ‘Leopard’:**

Linear model Poly22:

f(x,y) = p00 + p10*x + p01*y + p20*x^2 + p11*x*y + p02*y^2

Coefficients (with 95% confidence bounds):

p00 = -0.8475 (-1.317, -0.3778)

p10 = 4.233 (2.525, 5.94)

p01 = 0.2358 (-1.538, 2.01)

p20 = -3.98 (-5.557, -2.403)

p11 = 2.817 (0.2417, 5.393)

p02 = -2.695 (-4.272, -1.118)

General quintic equation for **cultivar ‘Leopard’**:

Linear model Poly55:

f(x,y) = p00 + p10*x + p01*y + p20*x^2 + p11*x*y + p02*y^2 + p30*x^3 + p21*x^2*y

+ p12*x*y^2 + p03*y^3 + p40*x^4 + p31*x^3*y + p22*x^2*y^2

+ p13*x*y^3 + p04*y^4 + p50*x^5 + p41*x^4*y + p32*x^3*y^2

+ p23*x^2*y^3 + p14*x*y^4 + p05*y^5

Coefficients (with 95% confidence bounds):

p00 = 0.7404 (-0.9528, 2.434)

p10 = 0.2894 (-0.113, 0.6919)

p01 = -0.5844 (-1.112, -0.05709)

p20 = -0.08301 (-0.1336, -0.0324)

p11 = 0.1236 (0.04183, 0.2054)

p02 = -0.003203 (-0.06525, 0.05884)

p30 = 0.005981 (0.003015, 0.008947)

p21 = -0.007083 (-0.01372, -0.0004443)

p12 = -0.001901 (-0.008736, 0.004934)

p03 = 0.001129 (-0.002225, 0.004484)

p40 = -0.0001659 (-0.0002449, -8.682e-005)

p31 = 0.0001543 (-7.969e-005, 0.0003882)

p22 = 0.0001408 (-0.0001927, 0.0004743)

p13 = -8.605e-005 (-0.0003246, 0.0001525)

p04 = -2.772e-006 (-8.646e-005, 8.091e-005)

p50 = 1.59e-006 (8.133e-007, 2.366e-006)

p41 = -1.193e-006 (-4.087e-006, 1.701e-006)

p32 = -2.022e-006 (-7.38e-006, 3.336e-006)

p23 = 9.495e-007 (-4.409e-006, 6.308e-006)

p14 = 4.962e-007 (-2.398e-006, 3.39e-006)

p05 = -1.364e-007 (-9.129e-007, 6.401e-007)

BP-ANN-Quintic equation for **cultivar ‘Leopard’:**

Linear model Poly55:

f(x,y) = p00 + p10*x + p01*y + p20*x^2 + p11*x*y + p02*y^2 + p30*x^3 + p21*x^2*y

+ p12*x*y^2 + p03*y^3 + p40*x^4 + p31*x^3*y + p22*x^2*y^2

+ p13*x*y^3 + p04*y^4 + p50*x^5 + p41*x^4*y + p32*x^3*y^2

+ p23*x^2*y^3 + p14*x*y^4 + p05*y^5

Coefficients (with 95% confidence bounds):

p00 = 1.172 (-0.275, 2.619)

p10 = 10.17 (-3.587, 23.92)

p01 = -28.48 (-46.5, -10.46)

p20 = -142.3 (-211.5, -73.1)

p11 = 252.2 (140.4, 364.1)

p02 = -17.41 (-102.2, 67.42)

p30 = 430.7 (268.5, 592.9)

p21 = -650.6 (-1014, -287.6)

p12 = -9.846 (-383.6, 363.9)

p03 = 38.42 (-145, 221.9)

p40 = -489.4 (-662.4, -316.5)

p31 = 639.8 (128, 1152)

p22 = 226.6 (-503, 956.1)

p13 = -246.8 (-768.5, 275)

p04 = 44.63 (-138.4, 227.7)

p50 = 189 (121, 256.9)

p41 = -201.4 (-454.7, 51.77)

p32 = -259.4 (-728.2, 209.5)

p23 = 344.3 (-124.5, 813.2)

p14 = -180.3 (-433.5, 72.92)

p05 = 48.98 (-18.97, 116.9)

General quadratic equation for **cultivar ‘Sapphire’:**

Linear model Poly22:

f(x,y) = p00 + p10*x + p01*y + p20*x^2 + p11*x*y + p02*y^2

Coefficients (with 95% confidence bounds):

p00 = -0.8695 (-1.329, -0.4101)

p10 = 0.1072 (0.0655, 0.149)

p01 = 0.006061 (-0.03732, 0.04945)

p20 = -0.002442 (-0.003406, -0.001477)

p11 = 0.001413 (-0.0001614, 0.002987)

p02 = -0.001385 (-0.002349, -0.0004212)

BP-ANN quadratic

Linear model Poly22:

f(x,y) = p00 + p10*x + p01*y + p20*x^2 + p11*x*y + p02*y^2

Coefficients (with 95% confidence bounds):

p00 = -0.8778 (-1.314, -0.4418)

p10 = 4.301 (2.716, 5.886)

p01 = 0.2724 (-1.375, 1.92)

p20 = -3.916 (-5.38, -2.451)

p11 = 2.261 (-0.1297, 4.652)

p02 = -2.245 (-3.71, -0.7813)

General quintic equation for **cultivar ‘Sapphire’:**

Linear model Poly55:

f(x,y) = p00 + p10*x + p01*y + p20*x^2 + p11*x*y + p02*y^2 + p30*x^3 + p21*x^2*y

+ p12*x*y^2 + p03*y^3 + p40*x^4 + p31*x^3*y + p22*x^2*y^2

+ p13*x*y^3 + p04*y^4 + p50*x^5 + p41*x^4*y + p32*x^3*y^2

+ p23*x^2*y^3 + p14*x*y^4 + p05*y^5

Coefficients (with 95% confidence bounds):

p00 = -0.2381 (-1.718, 1.242)

p10 = 0.4481 (0.0963, 0.7998)

p01 = -0.3614 (-0.8223, 0.09954)

p20 = -0.07896 (-0.1232, -0.03472)

p11 = 0.05086 (-0.02065, 0.1224)

p02 = 0.01664 (-0.03759, 0.07087)

p30 = 0.005446 (0.002854, 0.008038)

p21 = -0.004928 (-0.01073, 0.0008745)

p12 = 0.002589 (-0.003385, 0.008563)

p03 = -0.002317 (-0.005248, 0.0006153)

p40 = -0.0001544 (-0.0002235, -8.528e-005)

p31 = 0.0001623 (-4.218e-005, 0.0003668)

p22 = -7.678e-005 (-0.0003683, 0.0002147)

p13 = -4.181e-006 (-0.0002127, 0.0002043)

p04 = 4.943e-005 (-2.372e-005, 0.0001226)

p50 = 1.521e-006 (8.423e-007, 2.2e-006)

p41 = -1.691e-006 (-4.22e-006, 8.387e-007)

p32 = 3.126e-007 (-4.371e-006, 4.996e-006)

p23 = 1.016e-006 (-3.668e-006, 5.7e-006)

p14 = -7.56e-007 (-3.286e-006, 1.773e-006)

p05 = -1.592e-007 (-8.38e-007, 5.195e-007)

BP-ANN-quintic equation for **cultivar ‘Sapphire’:**

Linear model Poly55:

f(x,y) = p00 + p10*x + p01*y + p20*x^2 + p11*x*y + p02*y^2 + p30*x^3 + p21*x^2*y

+ p12*x*y^2 + p03*y^3 + p40*x^4 + p31*x^3*y + p22*x^2*y^2

+ p13*x*y^3 + p04*y^4 + p50*x^5 + p41*x^4*y + p32*x^3*y^2

+ p23*x^2*y^3 + p14*x*y^4 + p05*y^5

Coefficients (with 95% confidence bounds):

p00 = 0.223 (-1.283, 1.729)

p10 = 13.06 (-1.253, 27.38)

p01 = -15.93 (-34.69, 2.832)

p20 = -107.4 (-179.5, -35.41)

p11 = 97.4 (-19.01, 213.8)

p02 = 21.07 (-67.22, 109.4)

p30 = 314.7 (145.8, 483.5)

p21 = -377.2 (-755, 0.7096)

p12 = 216.1 (-173, 605.1)

p03 = -163.6 (-354.5, 27.32)

p40 = -371.5 (-551.5, -191.5)

p31 = 549.4 (16.71, 1082)

p22 = -426.5 (-1186, 332.9)

p13 = 178.1 (-364.9, 721.2)

p04 = 67.35 (-123.2, 257.9)

p50 = 151.1 (80.34, 221.8)

p41 = -254.2 (-517.8, 9.321)

p32 = 188.9 (-299.1, 676.9)

p23 = -7.434 (-495.5, 480.6)

p14 = -79.15 (-342.7, 184.4)

p05 = 5.622 (-65.1, 76.34)
